# Supplementary material for: Structures of complete HIV-1 TAR RNA portray a dynamic platform poised for protein binding and structural remodeling
Source: Nat Commun. 2025 Mar 6;16:2252. doi: 10.1038/s41467-025-57519-w (PMC11885821; doi:10.1038/s41467-025-57519-w)
Supplement: Supplementary file 1 — Supplementary Information [file 41467_2025_57519_MOESM1_ESM.pdf]

Supplementary Materials for:

Structures of complete HIV-1 TAR RNA portray a dynamic platform poised for protein binding and structural remodeling

Charles Bou-Nader, Katie A. Link, Krishna C. Suddala, Jay R. Knutson, and Jinwei Zhang.

Supplementary Tables 1-5

Supplementary Figures 1-10

RNA	IC_{50} (nM)
VA-I	11 ± 3
TAR^{2GC} (WT)	77 ± 10
$TAR^{\Delta 4bp}$	N.D.
$TAR^{\Delta 9bp}$	N.D.
$TAR^{\Delta 14bp}$	N.D.
$TAR^{\Delta 23\text{-}39}$	387 ± 11
$TAR^{\Delta C5/\Delta A17}$	90 ± 9
$TAR^{\Delta 23UCU25}$	417 ± 30
$TAR^{\Delta C5/\Delta A17/\Delta 23UCU25}$	16 ± 6
$TAR^{A48U/C49G/U50A}$	1683 ± 76

Supplementary Table 1. Summary of IC_{50} measurements in PKR inhibition assays.
Values are mean ± s.d. of n= 3 biologically independent replicates. N.D.: not determined due to weak inhibition.

Method	Sample in Cell	Titrant	K_d (μM)	Log (K_a)	ΔH (kcal.mol^{-1})
FP	VA-I	PKR	0.04 ± 0.002	7.4 ± 0.02	-
	TAR^{2GC} (WT)		0.035 ± 0.005	7.46 ± 0.06	
	TAR$^{\Delta 4bp}$		0.05 ± 0.003	7.34 ± 0.03	
	TAR$^{\Delta 9bp}$		0.13 ± 0.04	6.85 ± 0.11	
	TAR$^{\Delta 14bp}$		1.23 ± 1	6.03 ± 0.4	
	TAR$^{\Delta 23\text{-}39}$		0.054 ± 0.01	7.26 ± 0.01	
	TAR$^{\Delta C5/\Delta A17}$		0.06 ± 0.003	7.22 ± 0.02	
	TAR$^{\Delta 23UCU25}$		0.05 ± 0.007	7.34 ± 0.06	
	TAR $^{\Delta C5/\Delta A17/\Delta 23UCU25}$		0.02 ± 0.03	7.65 ± 0.01	
	TAR$^{A48U/C49G/U50A}$		0.02 ± 0.03	7.02 ± 0.11	
	VA-I	dsRBMs	0.22 ± 0.08	6.69 ± 0.18	
	TAR^{2GC} (WT)		0.12 ± 0.01	6.91 ± 0.04	
	TAR$^{\Delta 4bp}$		0.29 ± 0.02	6.54 ± 0.03	
	TAR$^{\Delta 9bp}$		1.9 ± 0.7	5.74 ± 0.17	
	TAR$^{\Delta 14bp}$		2.28 ± 0.7	5.65 ± 0.13	
	TAR$^{\Delta 23\text{-}39}$		0.19 ± 0.03	6.73 ± 0.06	
	TAR$^{\Delta C5/\Delta A17}$		0.12 ± 0.01	6.91 ± 0.04	
	TAR$^{\Delta 23UCU25}$		0.14 ± 0.01	6.86 ± 0.03	
	TAR $^{\Delta C5/\Delta A17/\Delta 23UCU25}$		0.07 ± 0.02	7.17 ± 0.13	
	TAR$^{A48U/C49G/U50A}$		0.36 ± 0.12	6.46 ± 0.17	
ITC	TAR^{2GC} (WT)	HIV-1 Tat$^{44\text{-}60}$ WT	0.16 ± 0.1	6.82 ± 0.2	-11.5 ± 1.7
	TAR^{GAAA}		0.6 ± 0.18	6.29 ± 0.14	-6.8 ± 0.7
	TAR$^{G21C/C41G}$		0.65 ± 0.2	6.21 ± 0.16	-9.6 ± 1
	TAR$^{A22U/U40A}$		0.67 ± 0.11	6.18 ± 0.08	-10.8 ± 0.6
	TAR^{U23C}		N.D.		
	TAR^{U23A}		N.D.		
	TAR^{C24U}		0.07 ± 0.07	7.3 ± 0.4	-11.4 ± 0.4
	TAR^{C24G}		0.1 ± 0.04	7.0 ± 0.16	-12.5 ± 0.6
	TAR^{C24A}		0.3 ± 0.1	6.5 ± 0.14	-10.6 ± 1.1
	TAR$^{\Delta U25}$		0.08 ± 0.01	7.13 ± 0.02	-12.3 ± 0.5
	TAR$^{\Delta 23UCU25}$		N.D.		
	TAR$^{G26C/C39G}$		N.D.		
	TAR$^{G28C/C37G}$		N.D.		
	Tat ^{R49A}	TAR^{2GC} WT	0.5 ± 0.2	6.3 ± 0.22	-9.05 ± 0.5
	Tat $^{K50A/K51A}$		0.63 ± 0.14	6.21 ± 0.1	-12.6 ± 0.3
	Tat ^{R52A}		69 ± 9	4.15 ± 0.07	-
	Tat ^{R55F}		0.64 ± 0.22	6.21 ± 0.15	-9.9 ± 0.43
	Tat ^{R55W}		0.95 ± 0.4	6.06 ± 0.23	-9.0 ± 1
	Tat ^{R56A}		0.49 ± 0.3	6.38 ± 0.32	-9.6 ± 2.1
	Tat $^{G48A/Q54A/Q60A}$		0.23 ± 0.01	6.65 ± 0.02	-12.6 ± 1.2
	Tat $^{G48P/Q54P/A58P}$		0.12 ± 0.02	6.91 ± 0.06	-10.7 ± 1.3

Supplementary Table 2. Summary of binding measurements using FP and ITC.
Values are mean ± s.d. of n= 3 biologically independent replicates. N.D.: not determined due to weak binding.

Method	RNA	$[Mg^{2+}]$ (mM)	T_{m1} (ºC)	T_{m2} (ºC)
DSC	TAR^{2GC}	2	78.2	83.0
	TAR^{3G}	2	79.0	-
	TAR^{3GC}	2	80.6	-
	TAR^{GAAA}	2	77.4	83.4
	$TAR^{G16A/A17G}$	2	79.8	-
	$TAR^{G16A/A17G/GAAA/ins59A}$	2	79.8	-
	TAR^{2GC}	0	70.9	78.8
	TAR^{3G}	0	72.4	-
	TAR^{3GC}	0	73.1	-
CD	TAR^{2GC}	0	71.0	-
	TAR^{3G}	0	70.6	-
	TAR^{3GC}	0	74.3	-
	TAR^{2GC}	2	77.9	-
	TAR^{3G}	2	76.5	-
	TAR^{3GC}	2	79.5	-

Supplementary Table 3. Summary of melting parameters by differential scanning calorimetry (DSC) and circular dichroism (CD).

	Full-length HIV-1 TAR-I (TAR^{GAAA})	Full-length HIV-1 TAR-II ($TAR^{G16A/A17G/GAAA/ins59A}$)	Full-length HIV-1 TAR-I + Tat^{44-60}	Full-length HIV-1 TAR-II + Ca^{2+}
**Data collection**				
Space group	$P2_1$	$P2_12_12$	$P2_1$	$P2_122_1$
Cell dimensions				
a, *b*, *c* (Å)	61.25, 41.98, 68.57	132.54, 32.58, 46.21	68.87, 40.51, 120.41	31.59, 47.26, 132.77
α, β, γ (°)	90, 93.44, 90	90, 90, 90	90, 94.61, 90	90, 90, 90
Resolution (Å) [a]	35.79- 2.35 (2.434 – 2.35)	33.13 – 2.25 (2.331 – 2.25)	40.01 – 2.749 (2.847 – 2.749)	44.53 – 2.761 (2.86 – 2.761)
R_{merge} [a]	0.051 (1.10)	0.135 (1.71)	0.238 (1.70)	0.23.0 (3.51)
$I / \sigma I$ [a]	14.38 (1.63)	7.98 (1.57)	4.89 (1.16)	13.82 (1.62)
$CC_{1/2}$ [a]	0.999 (0.868)	0.991 (0.442)	0.994 (0.711)	0.999 (0.669)
CC* [a]	1 (0.964)	0.998 (0.783)	0.999 (0.912)	1 (0.895)
Completeness (%) [a]	97.46 (97.92)	99.6 (98.69)	97.84 (99.14)	99.17 (95.32)
Redundancy [a]	6.6 (6.3)	7.1 (7.3)	7.4 (7.7)	42.8 (42.1)
**Refinement**				
Resolution (Å) [a]	35.79- 2.35 (2.434 – 2.35)	33.13 – 2.25 (2.331 – 2.25)	40.01 – 2.749 (2.847 – 2.749)	44.53 – 2.761 (2.86 – 2.761)
No. reflections [b]	97282 (8976)	70998 (7124)	1304418 (13383)	236082 (22373)
R_{work} / R_{free} [a]	0.263/0.282 (0.452/0.505)	0.194/0.222 (0.340/0.376)	0.249/0.288 (0.392/0.436)	0.244/0.294 (0.329/0.405)
No. atoms				
Macromolecule	2320	1188	4762	1174
Water	6	38	9	7
Ligands	66	32	131	1
Mean *B*-factors ($Å^2$)				
Macromolecule	100.19	54.02	58.14	67.04
Water	88.72	45.18	44.77	52.21
Ligands	99.11	109.16	67.49	62.51
R.m.s. deviations				
Bond lengths (Å)	0.004	0.006	0.002	0.002
Bond angles (°)	1.0	1.39	0.56	0.43
PDB accession code	9DE6	9DE7	9DE5	9DE8

Supplementary Table 4. Summary of crystallographic statistics.

[a] Values in parentheses are for the highest resolution shell.

[b] Values in parentheses are for the cross-validation set.

2AP position	Titrant	Titrant concentration	τ_1 (ns)	Amplitude (A1)	τ_2 (ns)	Amplitude (A2)	τ_3 (ns)	Amplitude (A3)	τ_{avg} (ns)
2AP3	-		0.57	0.55	3.16	0.29	8.96	0.15	2.57
	NC	1 µM	0.54	0.55	3.03	0.29	8.88	0.16	2.59
		2 µM	0.57	0.55	3.16	0.29	9.07	0.15	2.59
		5 µM	0.58	0.55	3.26	0.3	9.14	0.15	2.67
		10 µM	0.59	0.54	3.11	0.3	8.86	0.17	2.76
		20 µM	0.61	0.53	3.19	0.3	8.75	0.17	2.77
		30 µM	0.56	0.57	3.32	0.28	8.84	0.15	2.57
		40 µM	0.37	0.67	2.94	0.21	8.37	0.12	1.87
		50 µM	0.37	0.69	3	0.2	8.35	0.11	1.77
	dsRBMs	1 µM	0.62	0.56	3.06	0.3	8.62	0.15	2.56
		2 µM	0.57	0.58	3.02	0.29	8.75	0.13	2.34
		5 µM	0.54	0.58	2.99	0.29	8.84	0.14	2.41
		10 µM	0.49	0.57	2.86	0.28	8.84	0.15	2.41
		20 µM	0.46	0.57	2.83	0.27	8.94	0.16	2.46
		30 µM	0.52	0.55	3	0.28	9.03	0.17	2.66
		40 µM	0.3	0.74	2.74	0.17	9.03	0.1	1.6
		50 µM	0.22	0.84	2.64	0.1	8.92	0.06	1
	Tat	1 µM	0.54	0.55	3.03	0.29	8.88	0.16	2.60
		2 µM	0.57	0.55	3.16	0.29	9.07	0.15	2.59
		5 µM	0.58	0.55	3.26	0.3	9.14	0.15	2.67
		10 µM	0.59	0.54	3.11	0.3	8.86	0.17	2.76
		20 µM	0.61	0.53	3.19	0.3	8.75	0.17	2.77
		30 µM	0.56	0.57	3.32	0.28	8.84	0.15	2.57
		40 µM	0.37	0.67	2.95	0.21	8.37	0.12	1.87
		50 µM	0.37	0.69	3	0.2	8.35	0.11	1.77
	urea	8 M	0.78	0.45	3.17	0.48	7.65	0.07	2.41
2AP17	-		0.79	0.15	3.66	0.15	9.48	0.7	7.31
	NC	1 µM	0.69	0.17	3.66	0.18	9.25	0.65	6.79
		2 µM	0.71	0.19	3.79	0.2	9.09	0.61	6.44
		5 µM	0.64	0.21	3.42	0.21	8.99	0.57	5.98
		10 µM	0.53	0.27	3.23	0.21	8.79	0.52	5.39
		20 µM	0.65	0.27	3.51	0.22	89	0.5	5.4
		30 µM	0.63	0.27	3.52	0.22	8.72	0.51	5.39
		40 µM	0.63	0.29	3.54	0.23	8.78	0.49	5.3
		50 µM	0.62	0.29	3.66	0.23	8.81	0.48	5.25
	dsRBMs	1 µM	0.81	0.14	3.56	0.17	9.42	0.69	7.22
		2 µM	0.87	0.15	4.39	0.19	9.57	0.66	7.28
		5 µM	0.91	0.14	4	0.2	9.58	0.66	7.25
		10 µM	0.91	0.17	4.3	0.2	9.58	0.66	7.31
		20 µM	0.75	0.17	4.29	0.2	9.6	0.64	7.13
		30 µM	0.82	0.18	4.21	0.19	9.57	0.63	6.98
		40 µM	0.92	0.18	4.23	0.2	9.61	0.62	6.97
		50 µM	0.83	0.17	4.23	0.2	9.56	0.63	7.0
	Tat	1 µM	0.88	0.15	4.3	0.18	9.59	0.67	7.33
		2 µM	0.89	0.15	4.3	0.21	9.6	0.64	7.18
		5 µM	0.65	0.17	3.93	0.21	9.38	0.63	6.84
		10 µM	0.68	0.17	3.84	0.22	9.18	0.61	6.56
		20 µM	0.86	0.19	4.02	0.23	9.12	0.58	6.38
		30 µM	0.7	0.18	3.73	0.24	9.02	0.58	6.25
		40 µM	0.73	0.2	4.03	0.24	9.1	0.55	6.12
		50 µM	0.86	0.21	4.08	0.25	9.06	0.53	6.0
	urea	8 M	0.52	0.45	3.3	0.26	8.44	0.28	3.45
2AP20	-		0.33	0.33	0.7	0.17	6.62	0.13	1.41
	NC	1 µM	0.3	0.72	2.0	0.16	6.9	0.13	1.44

		2 µM	0.36	0.65	2.07	0.2	7.08	0.15	1.71
		5 µM	0.35	0.6	2.05	0.22	7.19	0.18	1.96
		10 µM	0.44	0.56	2.39	0.24	7.51	0.2	2.32
		20 µM	0.44	0.52	2.34	0.24	7.46	0.24	2.58
		30 µM	0.43	0.5	2.34	0.24	7.46	0.26	2.71
		40 µM	0.41	0.47	2.2	0.24	7.26	0.29	2.82
		50 µM	0.46	0.46	2.39	0.24	7.33	0.31	3.06
		1 µM	0.48	0.65	3.17	0.24	8.83	0.11	2.04
		2 µM	0.47	0.64	3.2	0.25	9	0.11	2.09
		5 µM	0.5	0.68	3.23	0.2	9	0.12	2.06
	dsRBMs	10 µM	0.5	0.63	3.23	0.25	9.11	0.12	2.21
		20 µM	0.45	0.64	3.26	0.25	9.37	0.11	2.14
		30 µM	0.52	0.62	3.64	0.27	9.83	0.11	2.39
		40 µM	0.49	0.62	3.63	0.27	10	0.11	2.38
		50 µM	0.5	0.61	3.67	0.28	10.2	0.11	2.45
		1 µM	0.26	0.74	1.75	0.15	6.57	0.11	1.18
		2 µM	0.28	0.73	1.81	0.15	6.6	0.11	1.2
		5 µM	0.24	0.75	1.75	0.14	6.62	0.11	1.15
	Tat	10 µM	0.25	0.74	1.83	0.15	6.67	0.11	1.19
		20 µM	0.16	0.88	1.82	0.07	6.66	0.05	0.6
		30 µM	0.04	0.98	1.52	0.01	6.24	0.01	0.12
		40 µM	0.1	0.94	1.55	0.04	6.09	0.02	0.27
		50 µM	0.06	0.97	1.57	0.02	6.08	0.01	0.15
	urea	8 M	0.54	0.54	2.74	0.22	5.3	0.24	2.17
	-		0.73	0.34	3.33	0.26	8.27	0.4	4.42
		1 µM	0.69	0.35	3.45	0.26	8.47	0.39	4.44
		2 µM	0.75	0.33	3.43	0.25	8.5	0.42	4.67
		5 µM	0.8	0.26	3.56	0.25	8.75	0.49	5.38
	NC	10 µM	0.73	0.2	3.74	0.26	9.08	0.55	6.11
		20 µM	0.72	0.18	3.39	0.22	8.95	0.6	6.24
		30 µM	0.92	0.2	3.83	0.24	9.05	0.56	6.17
		40 µM	0.8	0.21	3.33	0.24	8.88	0.55	5.85
		50 µM	0.83	0.25	3.55	0.26	8.93	0.49	5.51
		1 µM	0.8	0.28	3.72	0.27	8.9	0.45	5.23
		2 µM	0.66	0.28	3.52	0.28	9.06	0.45	5.25
		5 µM	0.88	0.25	3.9	0.27	9.31	0.48	5.74
2AP24	dsRBMs	10 µM	0.68	0.24	3.64	0.26	9.34	0.5	5.9
		20 µM	0.68	0.24	3.8	0.26	9.5	0.5	5.9
		30 µM	0.8	0.21	3.79	0.26	9.45	0.53	6.16
		40 µM	0.78	0.21	3.81	0.27	9.52	0.52	6.14
		50 µM	0.77	0.21	3.81	0.26	9.52	0.52	6.1
		1 µM	0.76	0.3	3.58	0.26	8.7	0.44	4.99
		2 µM	0.73	0.28	3.5	0.27	8.85	0.45	5.13
		5 µM	0.84	0.22	3.81	0.27	9.3	0.51	5.96
	Tat	10 µM	0.74	0.18	3.77	0.27	9.34	0.55	6.29
		20 µM	0.77	0.18	3.92	0.27	9.43	0.55	6.38
		30 µM	0.87	0.17	4.07	0.28	9.5	0.55	6.51
		40 µM	0.75	0.18	3.91	0.28	9.42	0.55	6.41
		50 µM	0.86	0.19	4.14	0.3	9.42	0.51	6.21
	urea	8 M	0.75	0.39	2.8	0.28	4.87	0.32	2.63

Supplementary Table 5. Summary of representative 2-aminopurine (2AP) fluorescence lifetime measurements.

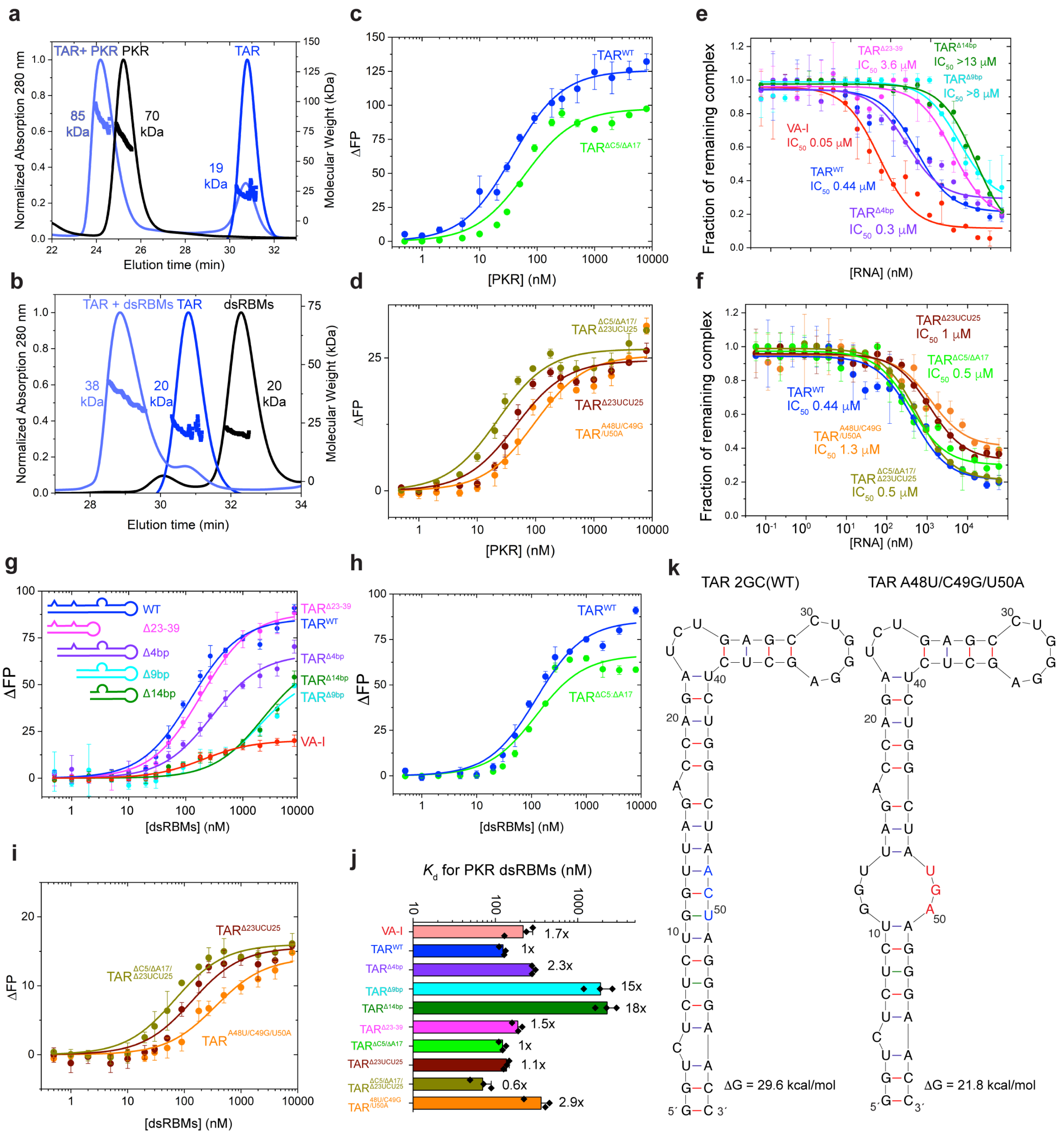

a
TAR+ PKR
PKR
TAR
85 kDa
70 kDa
19 kDa
Normalized Absorption 280 nm
Molecular Weight (kDa)
Elution time (min)
b
TAR + dsRBMs
TAR
dsRBMs
38 kDa
20 kDa
20 kDa
c
ΔFP
[PKR] (nM)
d
e
Fraction of remaining complex
[RNA] (nM)
VA-I IC50 0.05 μM
TAR IC50 0.44 μM
TAR IC50 3.6 μM
TAR IC50 >13 μM
TAR IC50 >8 μM
TAR IC50 0.3 μM
f
TAR IC50 1 μM
TAR IC50 0.5 μM
TAR IC50 0.44 μM
TAR IC50 1.3 μM
TAR IC50 0.5 μM
g
WT
Δ23-39
Δ4bp
Δ9bp
Δ14bp
VA-I
[dsRBMs] (nM)
h
i
j
Kd for PKR dsRBMs (nM)
1.7x
1x
2.3x
15x
18x
1.5x
1x
1.1x
0.6x
2.9x
k
TAR 2GC(WT)
TAR A48U/C49G/U50A
ΔG = 29.6 kcal/mol
ΔG = 21.8 kcal/mol

Supplementary Fig. 1 | SEC-MALS and FP analyses of TAR interactions with PKR and its dsRBMs.

a Size exclusion chromatography coupled to multi-angle light scattering (SEC-MALS) profiles of full-length PKR (black), HIV-1 TAR (dark blue) and stoichiometric (1:1) mixture of PKR with TAR (light blue). SEC-MALS-derived molecular weights are indicated. **b** SEC-MALS profiles of dsRBMs of PKR (black), TAR (dark blue) and stoichiometric (1:1) mixture of dsRBMs with TAR (light blue). **c, d** Fluorescence polarization (FP) titrations of WT and variant TAR RNAs with full-length PKR. **e, f** Competitive binding experiments where fluorescently labeled WT TAR pre-bound to PKR were challenged by increasing amounts of unlabeled 79-bp dsRNA competitor. Apparent IC_{50}s are indicated. Values are mean ± s.d., n=3 biologically independent samples. **g-i** Fluorescence polarization titrations of VA-I and TAR variants with increasing concentrations of dsRBMs of PKR. **j** Comparisons of dsRBMs-RNA binding affinities obtained from g-i. **k** Mfold[1] analyses of the predicted secondary structures of the WT TAR^{2GC} (left) and A48U/C49G/U50A mutant TAR. The original and new trinucleotides are indicated in blue and red, respectively. Theoretical free energies of RNA folding are also indicated.

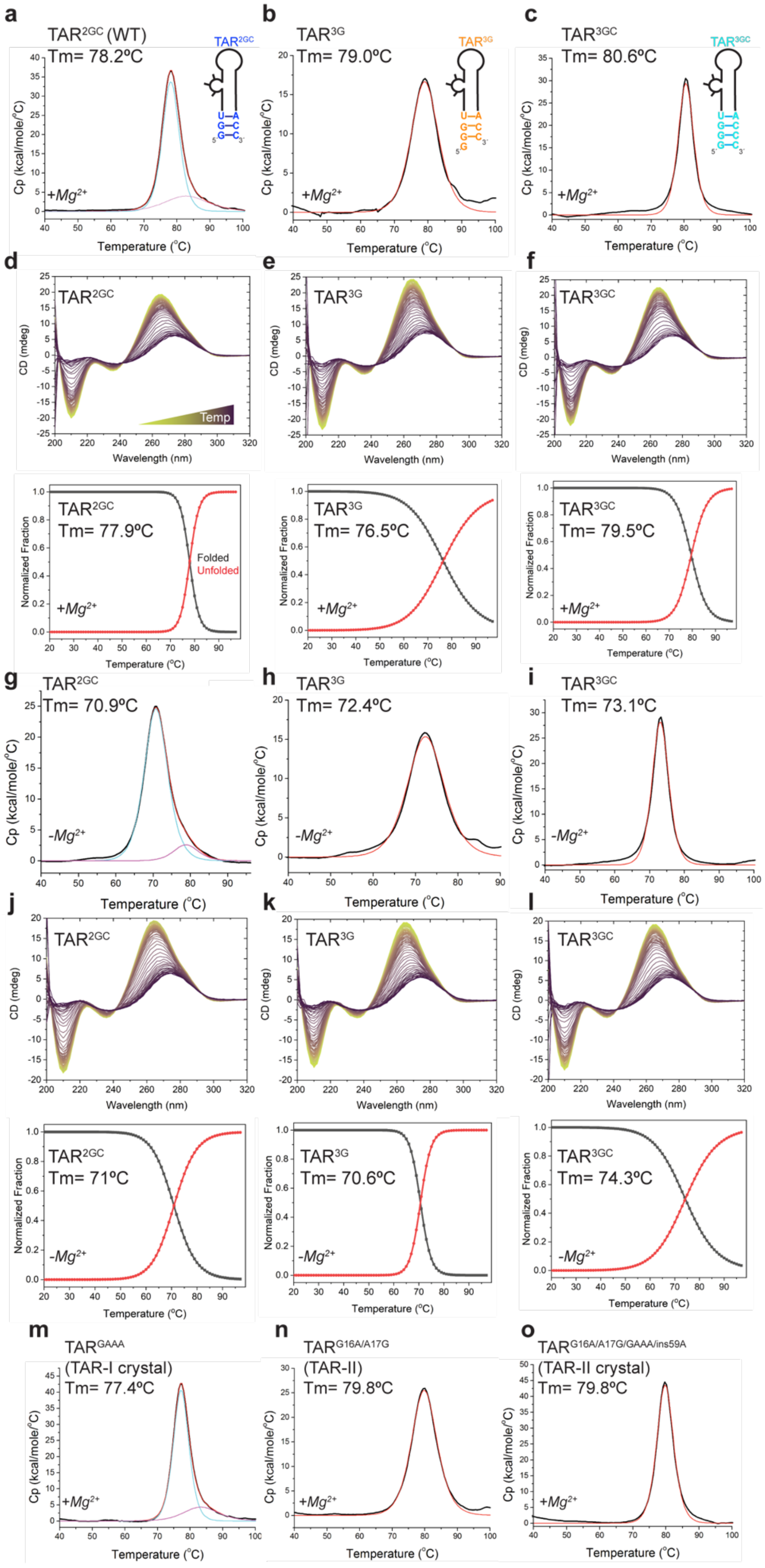

a
TAR2GC (WT)
Tm= 78.2ºC
+Mg2+
b
TAR3G
Tm= 79.0ºC
+Mg2+
c
TAR3GC
Tm= 80.6ºC
+Mg2+
Cp (kcal/mole/ºC)
Temperature (ºC)
d
TAR2GC
Temp
e
TAR3G
f
TAR3GC
CD (mdeg)
Wavelength (nm)
TAR2GC
Tm= 77.9ºC
Folded
Unfolded
+Mg2+
TAR3G
Tm= 76.5ºC
+Mg2+
TAR3GC
Tm= 79.5ºC
+Mg2+
Normalized Fraction
Temperature (ºC)
g
TAR2GC
Tm= 70.9ºC
-Mg2+
h
TAR3G
Tm= 72.4ºC
-Mg2+
i
TAR3GC
Tm= 73.1ºC
-Mg2+
j
TAR2GC
k
TAR3G
l
TAR3GC
TAR2GC
Tm= 71ºC
-Mg2+
TAR3G
Tm= 70.6ºC
-Mg2+
TAR3GC
Tm= 74.3ºC
-Mg2+
m
TARGAAA
(TAR-I crystal)
Tm= 77.4ºC
+Mg2+
n
TARG16A/A17G
(TAR-II)
Tm= 79.8ºC
+Mg2+
o
TARG16A/A17G/GAAA/ins59A
(TAR-II crystal)
Tm= 79.8ºC
+Mg2+

Supplementary Fig. 2 | DSC and CD analyses of WT and variant TAR thermostabilities.
a-c DSC profiles of $TAR^{2GC\ (WT)}$ (**a**), TAR^{3G} (**b**) and TAR^{3GC} (**c**) in the presence of 2 mM Mg^{2+}. The measured melting temperatures (T_m) are indicated. **d-f** Upper panels: temperature-scanning CD profiles of TAR^{2GC} (**d**), TAR^{3G} (**e**) and TAR^{3GC} (**f**) collected from 20 to 97°C in 1°C intervals in the presence of 2 mM Mg^{2+}. Lower panels: fractions of folded (black) and unfolded (red) TAR as functions of temperature derived by global fitting of the CD profiles above. **g-i** DSC profiles of TAR^{2GC} (**g**), TAR^{3G} (**h**) and TAR^{3GC} (**i**) in the absence of Mg^{2+}. **j-l** Upper panels: temperature-scanning CD profiles of TAR^{2GC} (**j**), TAR^{3G} (**k**) and TAR^{3GC} (**l**) collected from 20 to 97°C in 1°C intervals in the absence of Mg^{2+}. Lower panels: fractions of folded (black) and unfolded (red) TAR as functions of temperature derived by global fitting of the CD profiles above. **m-o** DSC analyses in the presence of 2 mM Mg^{2+} of TAR^{GAAA} (**m**), $TAR^{G16A/A17G}$ (**n**), and $TAR^{G16A/A17G/GAAA/ins59A}$ (**o**). The measured melting temperatures (T_m) are indicated. Experiments performed once.

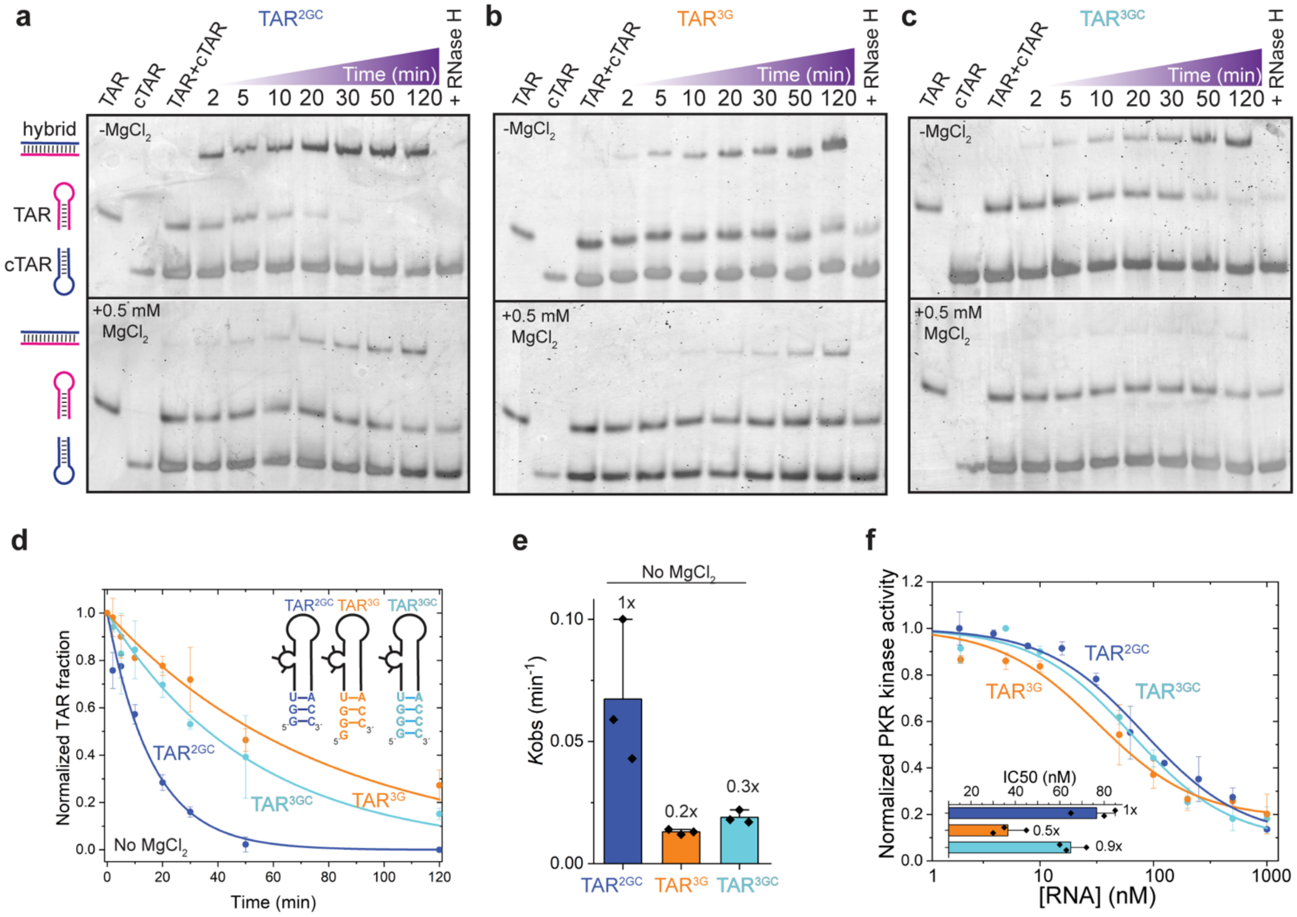

Supplementary Fig. 3 | Effects of TAR 5′ end heterogeneity on TAR-cTAR annealing and PKR inhibition.

a-c Representative non-denaturing PAGE analyses of NC-mediated annealing of cTAR to TAR^{2GC} (**a**), TAR^{3G} (**b**), and TAR^{3GC} (**c**) in the absence (upper panels) or presence (lower panels) of 0.5 mM Mg^{2+}. **d** Plot of the remaining TAR RNA fractions as functions of time in the absence of Mg^{2+} in (a-c). **e** Comparison of the apparent rates of annealing derived from the data in d. **f** PKR-inhibition profiles by TAR^{2GC} (blue), TAR^{3G} (orange) and TAR^{3GC} (cyan). Insert: the derived IC_{50} values. Values are mean ± s.d. of 3 biologically independent replicates.

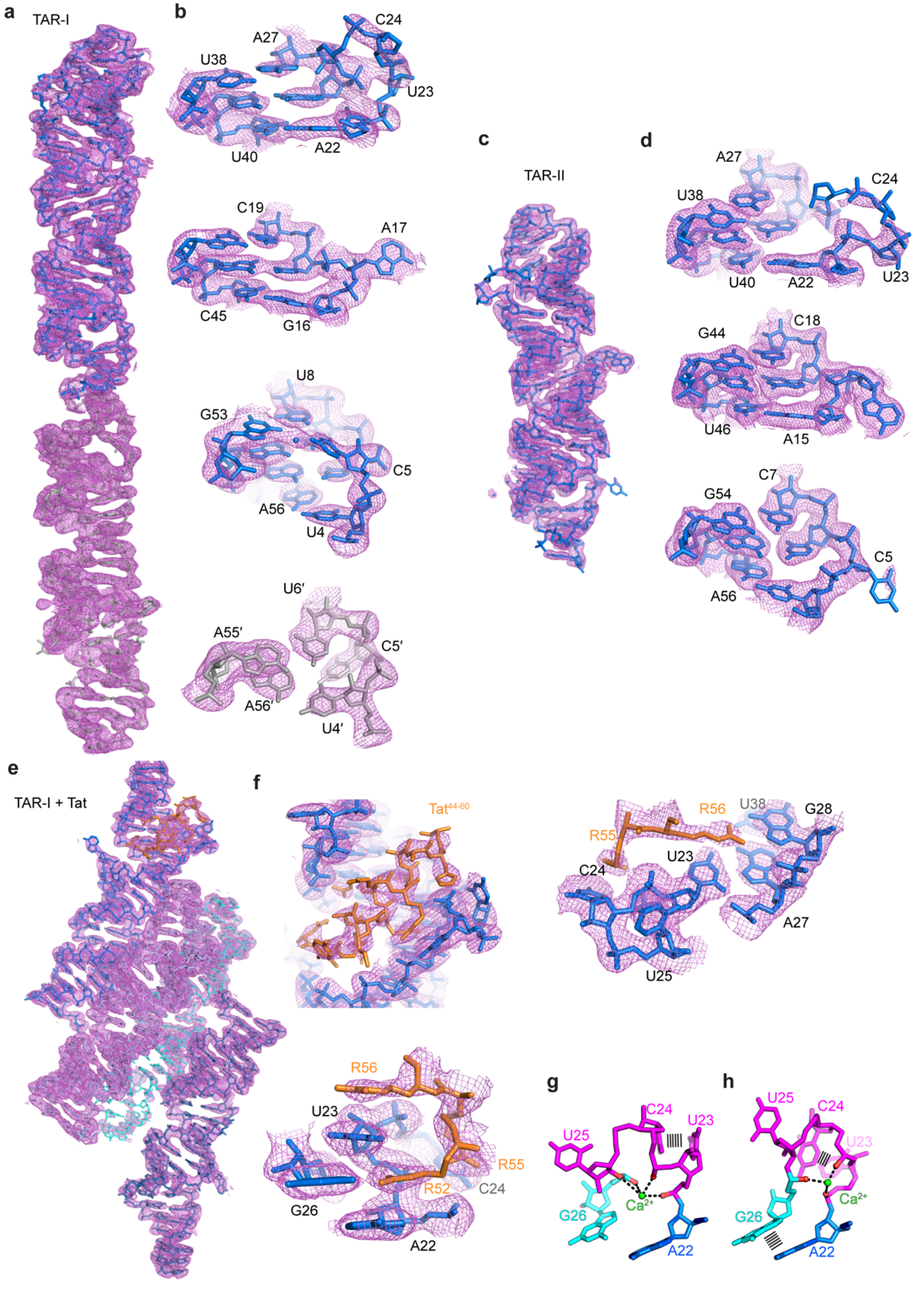
a
TAR-I
b
C24
A27
U38
U23
U40
A22
C19
A17
C45
G16
U8
G53
C5
A56
U4
U6′
A55′
C5′
A56′
U4′
c
TAR-II
d
A27
C24
U38
U40
A22
U23
C18
G44
U46
A15
C7
G54
C5
A56
e
TAR-I + Tat
f
Tat44-60
R56
U38
G28
R55
U23
C24
A27
U25
R56
U23
R55
R52
C24
G26
A22
g
U25
C24
U23
G26
Ca2+
A22
h
U25
C24
U23
G26
Ca2+
A22

Supplementary Fig. 4 | Representative electron densities for TAR-I, TAR-II, and Tat-bound TAR-I crystal structures. a Composite simulated anneal-omit 2Fo-Fc electron densities calculated using the final model and superimposed with the final refined model contoured at 1σ for TAR-I. **b** Four portions of the map in a. Apostrophes denote symmetry-related molecules. **c** Composite simulated anneal-omit 2Fo-Fc electron densities calculated using the final model and superimposed with the final refined model contoured at 1σ for TAR-II. **d** Three portions of the map in c. **e** Composite simulated anneal-omit 2Fo-Fc electron densities calculated using the final model and superimposed with the final refined model contoured at 1σ for TAR-I (blue and cyan) soaked with HIV-1 Tat^{44-60} (orange). **f** Three portions of the map in e**. g** Structure of the UCU bulge in TAR-II soaked with $CaCl_2$. **h** Structure of the UCU bulge in the mini-TAR crystal structure soaked with $CaCl_2$ (PDB:397D)[2].

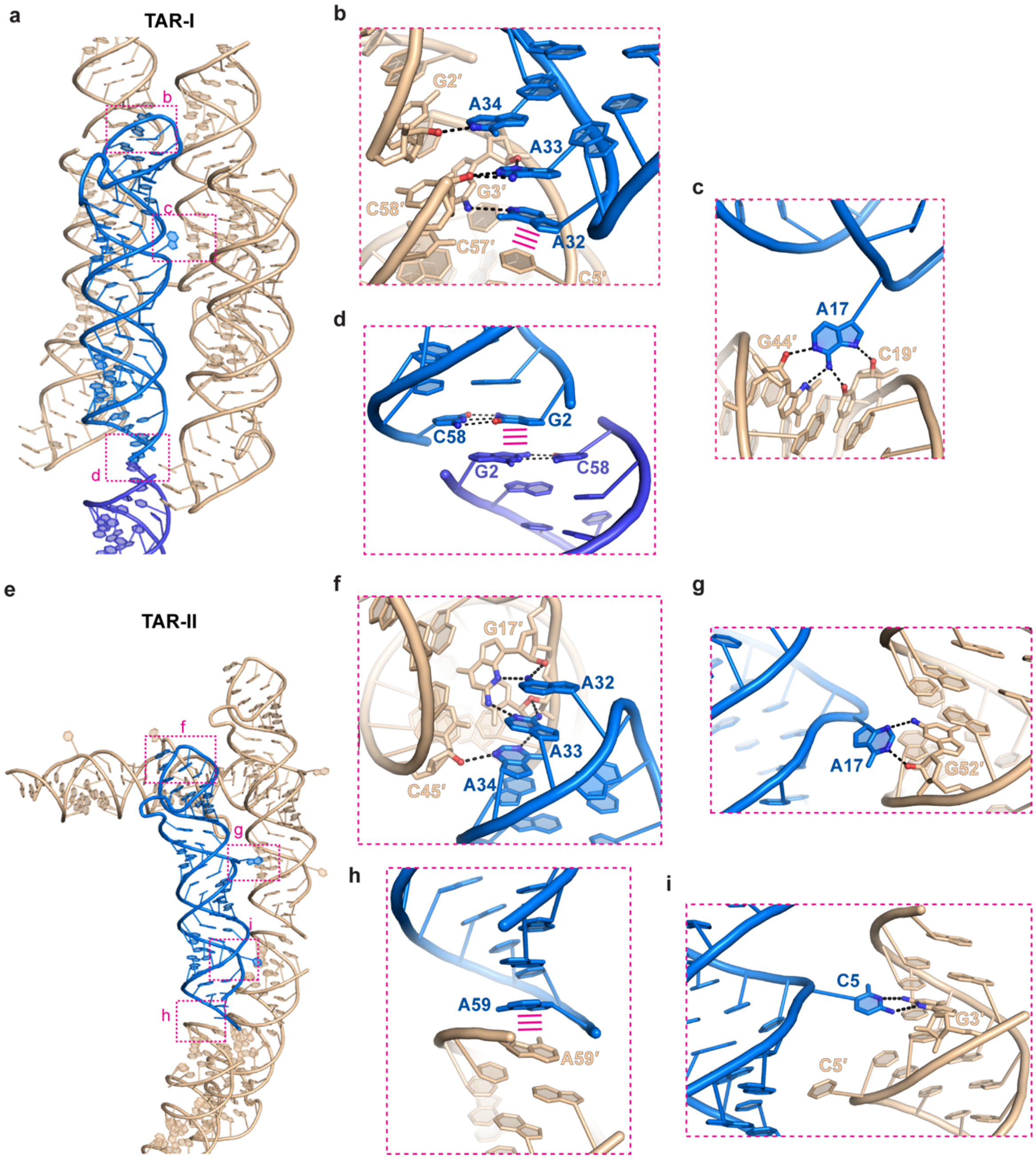

Supplementary Fig. 5 | Crystal-packing arrangements and interfaces in TAR crystals. a Overall crystal-packing arrangements in TAR-I crystals. The reference and symmetry-related molecules are shown in blue and wheat, respectively. **b-d** Detailed crystal-packing interfaces and contacts in the boxes in a. Black dotted lines represent hydrogen bonds and magenta lines indicate stacking interactions. Apostrophes denote symmetry-related molecules. **e** Overall crystal-packing arrangements in TAR-II crystals. **f-i** Detailed crystal-packing interfaces and contacts in the boxes in e. Apostrophes denote symmetry-related molecules.

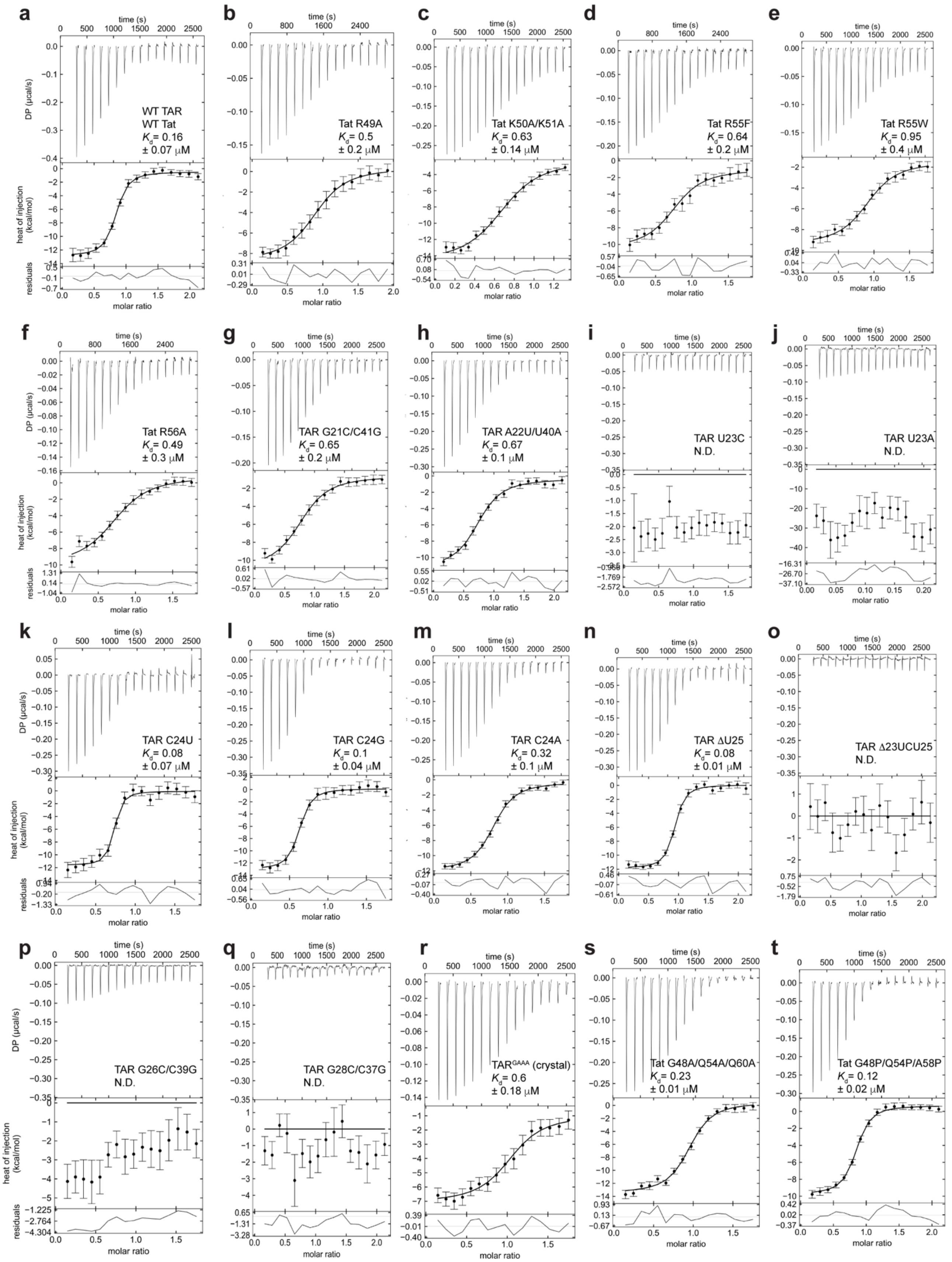

a
WT TAR
WT Tat
K_d= 0.16
± 0.07 μM
b
Tat R49A
K_d= 0.5
± 0.2 μM
c
Tat K50A/K51A
K_d= 0.63
± 0.14 μM
d
Tat R55F
K_d= 0.64
± 0.2 μM
e
Tat R55W
K_d= 0.95
± 0.4 μM
f
Tat R56A
K_d= 0.49
± 0.3 μM
g
TAR G21C/C41G
K_d= 0.65
± 0.2 μM
h
TAR A22U/U40A
K_d= 0.67
± 0.1 μM
i
TAR U23C
N.D.
j
TAR U23A
N.D.
k
TAR C24U
K_d= 0.08
± 0.07 μM
l
TAR C24G
K_d= 0.1
± 0.04 μM
m
TAR C24A
K_d= 0.32
± 0.1 μM
n
TAR ΔU25
K_d= 0.08
± 0.01 μM
o
TAR Δ23UCU25
N.D.
p
TAR G26C/C39G
N.D.
q
TAR G28C/C37G
N.D.
r
TAR^{GAAA} (crystal)
K_d= 0.6
± 0.18 μM
s
Tat G48A/Q54A/Q60A
K_d= 0.23
± 0.01 μM
t
Tat G48P/Q54P/A58P
K_d= 0.12
± 0.02 μM
time (s)
DP (μcal/s)
heat of injection (kcal/mol)
residuals
molar ratio

Supplementary Fig. 6 | Representative ITC isotherms for HIV-1 TAR binding to HIV-1 $Tat^{44\text{-}60}$. a-f Representative ITC isotherms for $TAR^{2GC(WT)}$ binding to WT (**a**), R49A (**b**), K50A/K51A (**c**), R55F (**d**), R55W (**e**) and R56A (**f**) $Tat^{44\text{-}60}$ variants. **g-r** Representative ITC isotherms for $Tat^{44\text{-}60}$ binding to G21C/C41G (**g**), A22U/U40A (**h**), U23C (**i**), U23A (**j**), C24U (**k**), C24G (**l**), C24A (**m**), ΔU25 (**n**), ΔU23UCU25 (**o**), G26C/C39G (**p**), G28C/C37G (**q**) and GAAA (**r**) TAR constructs. **s-t** Representative ITC isotherms for $TAR^{2GC(WT)}$ binding to G48A/Q54A/Q60A (**s**) and G48P/Q54P/A58P (**t**) $Tat^{44\text{-}60}$ variants. The constructs names and the K_d values (mean ± s.d., n=3 biologically independent replicates) are indicated in the top panels of the ITC isotherms.

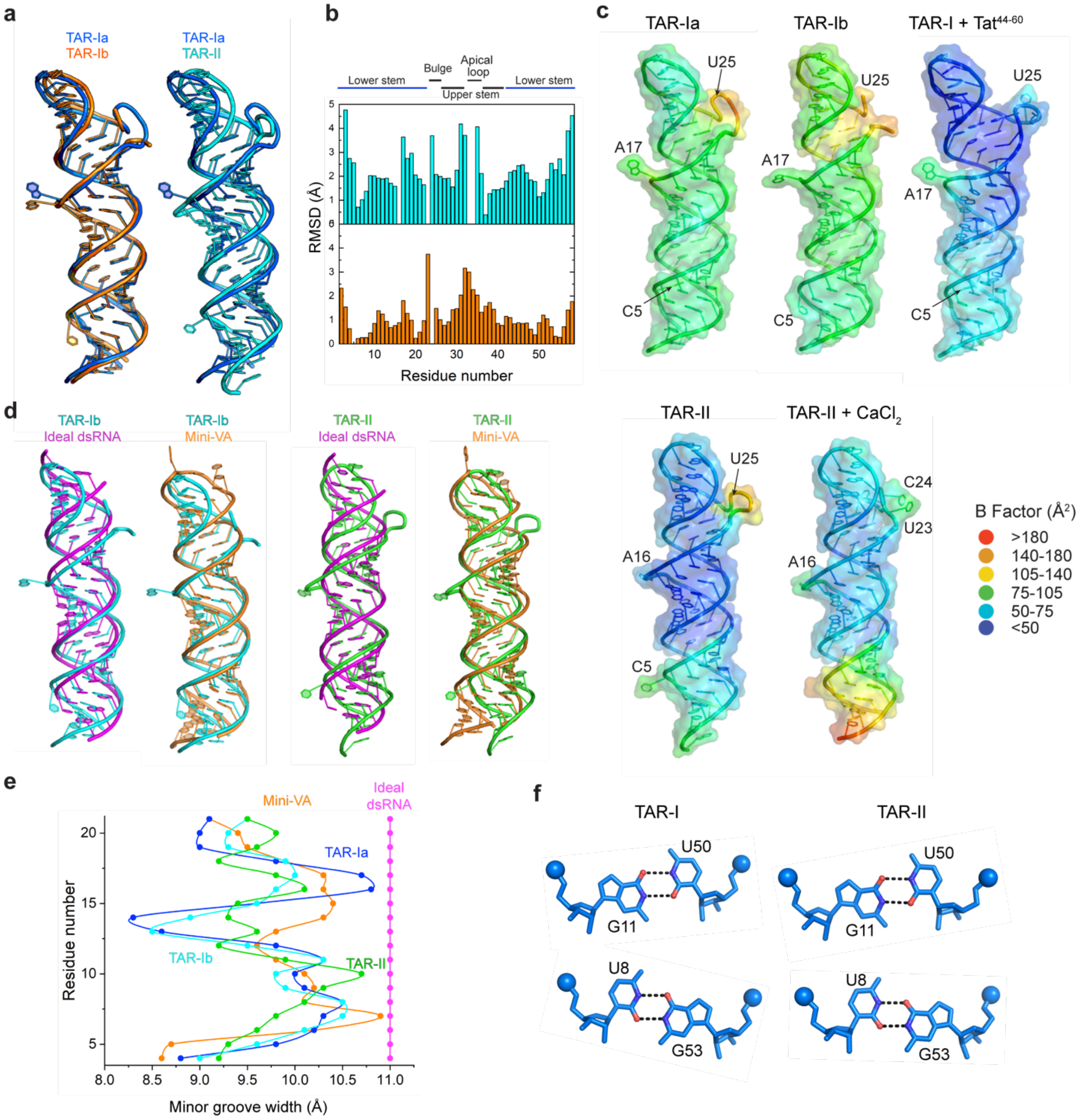

Supplementary Fig. 7 | Comparison of the TAR crystal structures. a Structural overlay of TAR-Ib (orange) and TAR-II (cyan) onto TAR-Ia (blue). **b** Pair-wise root mean square deviations (RMSDs) per residue from a, colored as in a. **c** *B*-factor distributions of TAR structures. **d** Structural overlay of free TAR structures with an ideal A-form dsRNA (magenta) or mini-VA (the fused apical stem-tetrastem of VA-I RNA, orange; PDB: 6OL3[3]). **e** Minor groove widths of RNAs shown in d. **f** G•U wobble base pairs observed in TAR-I (left) and TAR-II (right) crystal structures.

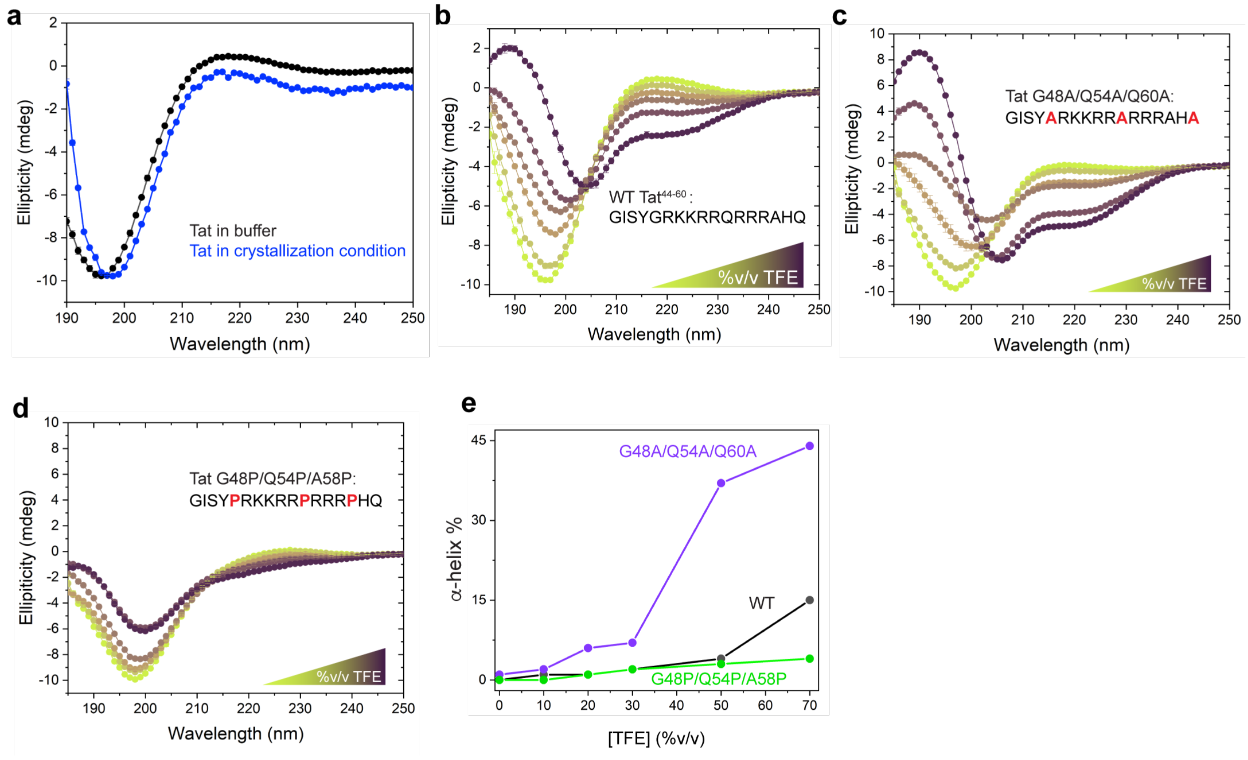

Supplementary Fig. 8 | CD analyses of WT and variant Tat peptides.
a CD spectra of WT Tat$^{44\text{-}60}$ in CD buffer (black, 25 mM Tris-HCl pH 7.5 and 25 mM NaCl) or crystallization condition (blue, 25 mM sodium cacodylate pH 6.5, 25 mM NaCl and 50% v/v 2-methyl-2,4-pentanediol). **b-d** CD spectra of WT (**b**), G48A/Q54A/Q60A (**c**), and G48P/Q54P/A58P (**d**) Tat$^{44\text{-}60}$ peptides in the presence of increasing concentrations of 2,2,2-Trifluoroethanol (TFE). Peptide sequences are indicated with mutations shown in red. **e** Plot of α-helix percentage in WT and variant Tat$^{44\text{-}60}$ in the presence of increasing TFE concentration, derived from CD data in b-d. Experiments performed once.

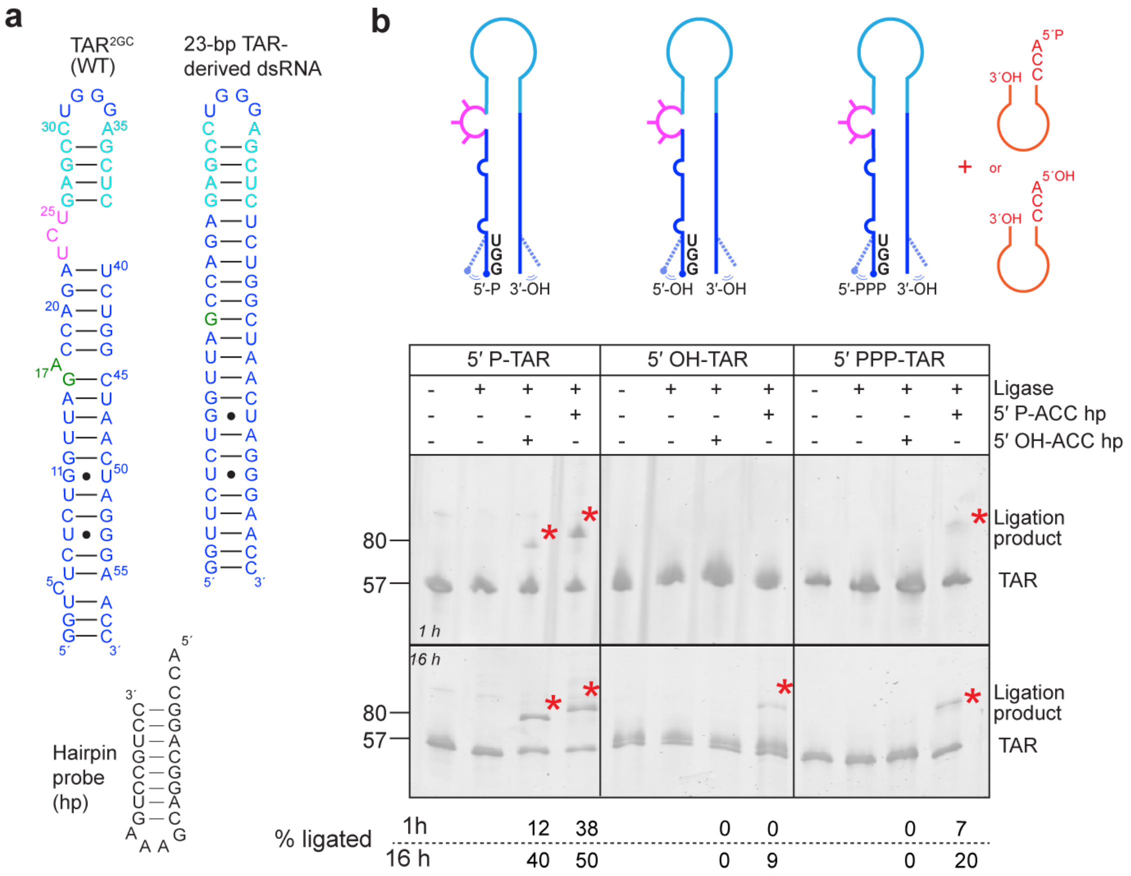

Supplementary Fig. 9 | Additional validation of the RHOALO assay. a Secondary structures of TAR^{2GC} (WT), a TAR-derived 23-bp dsRNA lacking bulged nucleotides, and a hairpin probe used in RHOALO. **b** Cartoon schematics and Urea-PAGE analyses of RHOALO ligation reactions performed using TAR^{2GC} RNAs that harbor 5′ monophosphate, 5′ triphosphate, or 5′ OH groups, and hairpin probes that contain 5′ monophosphate or 5′ OH groups, showing the requirement of 5′-monophosphates for efficient ligation. Red asterisks indicate the ligation products. hp: hairpin. The RNA lengths are indicated in nucleotides. Ligation efficiency is indicated below the gels. Experiments performed once.

.

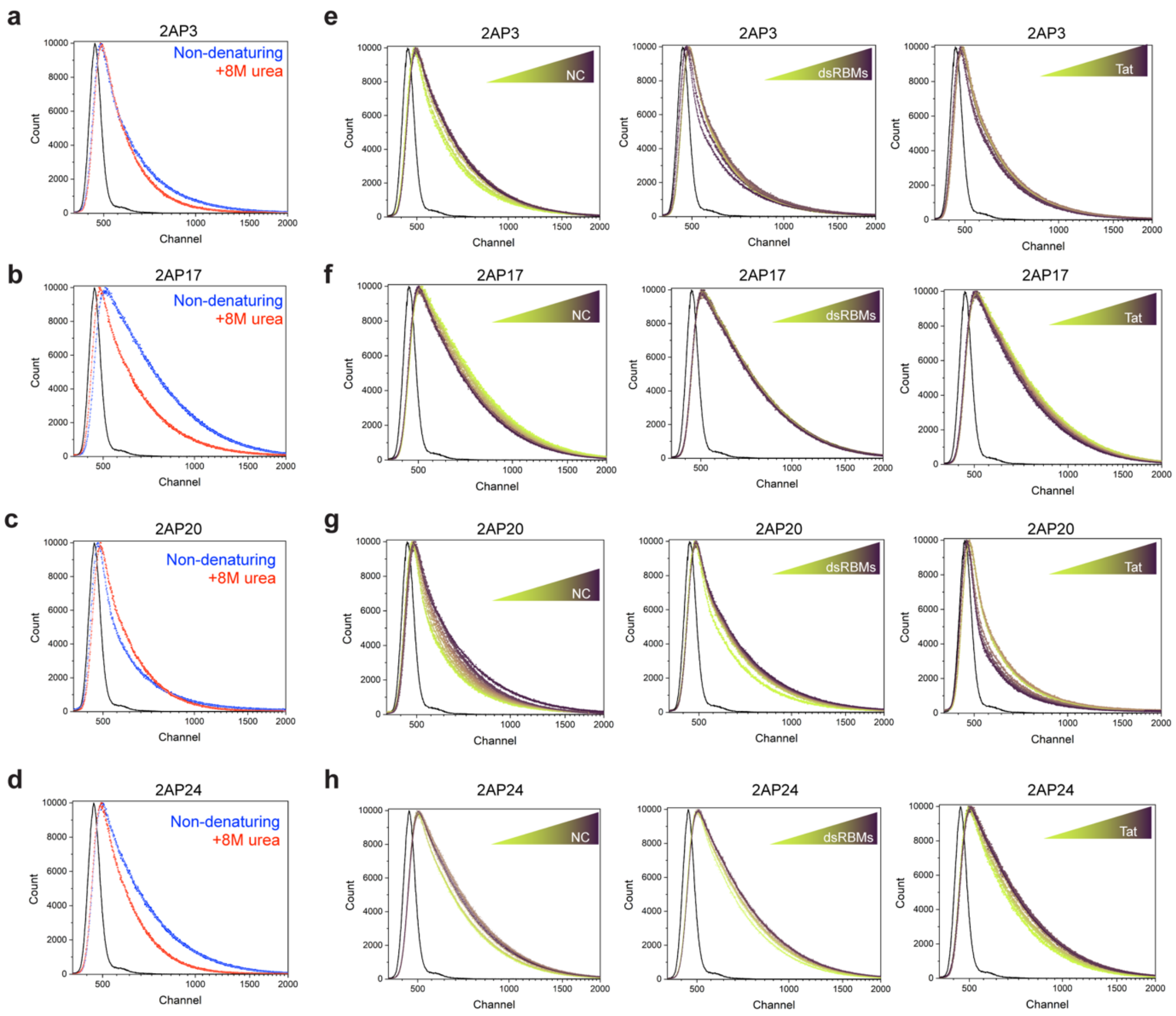

Supplementary Fig. 10 | 2AP TCSPC (Time Correlated Single Photon Counting) time traces. a-d Representative time-resolved fluorescence intensity decay profiles of 2AP at position 3 (**a**), position 17 (**b**), position 20 (**c**) and position 24 (**d**) in free TAR^{2GC}, under non-denaturing conditions (blue) or in 8M urea (red). **e-h** Representative time-resolved fluorescence intensity decay profiles of 2AP at position 3 (**e**), position 17 (**f**), position 20 (**g**) and position 24 (**h**) of TAR^{2GC} in the presence of increasing concentrations (from green to brown) of NC (left), dsRBMs of PKR (middle) or Tat (right). n=3 biologically independent replicates.

Supplementary References:

1. Zuker, M. Mfold web server for nucleic acid folding and hybridization prediction. *Nucleic Acids Res* **31**, 3406-15 (2003).
2. Ippolito, J.A. & Steitz, T.A. A 1.3-A resolution crystal structure of the HIV-1 trans-activation response region RNA stem reveals a metal ion-dependent bulge conformation. *Proc Natl Acad Sci U S A* **95**, 9819-24 (1998).
3. Hood, I.V. et al. Crystal structure of an adenovirus virus-associated RNA. *Nat Commun* **10**, 2871 (2019).
